# A cohort analysis to identify eligible patients for intraoperative radiotherapy (IORT) of early breast cancer

**DOI:** 10.1186/1748-717X-9-154

**Published:** 2014-07-12

**Authors:** Elena Sperk, Daniela Astor, Anke Keller, Grit Welzel, Axel Gerhardt, Benjamin Tuschy, Marc Sütterlin, Frederik Wenz

**Affiliations:** 1 Department of Radiation Oncology, University Medical Center Mannheim, University of Heidelberg, Theodor-Kutzer-Ufer 1-3, Mannheim, 68167, Germany; 2 Department of Obstetrics and Gynecology, University Medical Center Mannheim, University of Heidelberg, Theodor-Kutzer-Ufer 1-3, Mannheim, 68167, Germany

**Keywords:** Intraoperative radiotherapy, IORT, Breast cancer, APBI, Boost, Eligibility, Suitable patients

## Abstract

**Background:**

Since the results from the randomized TARGIT A trial were published, intraoperative radiotherapy (IORT) is used more often. IORT can be provided as accelerated partial breast irradiation (APBI) or as a boost. The definition of suitable patients for IORT as APBI differs between different national societies (e.g. ESTRO and ASTRO) and different inclusion criteria of trials and so does the eligibility of patients. This analysis identifies eligible patients for IORT according to available consensus statements and inclusion criteria of the ongoing TARGIT trials.

**Methods:**

Between 01/03 – 12/09, 1505 breast cancer cases were treated at the breast cancer center at the University Medical Center Mannheim. Complete data sets for age, stage (T, N, and M), histology and hormone receptor status were available in 1108 cases. Parameters to identify eligible patients are as follows: *ESTRO:* >50 years, invasive ductal carcinoma/other favorable histology (IDC), T1-2 (≤3 cm), N0, any hormone receptor status, M0; *ASTRO:* ≥60 years, IDC, T1, N0, positive estrogen hormone receptor status, M0; *TARGIT E “elderly*”, risk adapted radiotherapy with IORT followed by external beam radiotherapy in case of risk factors in final histopathology, phase II: ≥70 years, IDC, T1, N0, any hormone receptor status, M0; *TARGIT C “consolidation”*, risk adapted radiotherapy, phase IV: ≥50 years, IDC, T1, N0, positive hormone receptor status, M0; *TARGIT BQR “boost quality registry”*: every age, every histology, T1-2 (max. 3.5 cm), any hormone receptor status, N0/+, M0/+.

**Results:**

Out of the 1108 cases, 379 cases (34.2%) were suitable for IORT as APBI regarding the ESTRO and 175 (15.8%) regarding the ASTRO consensus statements. 82 (7.4%) patients were eligible for the TARGIT E trial, 258 (23.3%) for the TARGIT C trial and 671 (60.6%) for the TARGIT BQR registry. According to the consensus statements of ASTRO (45.1%) and ESTRO (41.4%) about half of the eligible patients were treated with IORT as APBI. From the eligible patients fulfilling the criteria for IORT boost (35%) about one third was eventually treated.

**Conclusions:**

Patient selection for IORT should be restrictive. For IORT as APBI the TARGIT trials are even more restrictive including patients than the ESTRO and ASTRO consensus statements.

## Background

Accelerated partial breast irradiation (APBI) has been reported having comparable local control and an excellent cosmetic outcome in comparison to whole breast radiotherapy using different techniques such as balloon brachytherapy [1], multi-catheter technique [2], external beam accelerated partial breast irradiation [3], intraoperative radiotherapy (IORT) with electrons (=IOERT) [4,5] or with low energy x-rays (=LEX-IORT) [6,7]. LEX-IORT with 50 kV x-rays is more often used since the results from the TARGIT A trial showed a non-inferiority regarding local control [6,7], less toxicity, especially chronic skin toxicity [8], and an overall survival benefit [7] for patients treated with LEX-IORT in comparison to patients treated with external beam radiotherapy (EBRT). The TARGIT A trial closed recruitment in June 2012. In the meantime the TARGIT E(lderly), TARGIT C(onsolidation) and the TARGIT B(oost)Q(uality)R(egistry) have been conducted or are ongoing. The TARGIT E trial is a phase II, non-randomized single arm trial for elderly patients above the age of 70 years (ClinicalTrials.gov Identifier: NCT01299987) [9]. In this trial all patients receive LEX-IORT without randomization. EBRT to the whole breast is only added after LEX-IORT, if risk factors are present in final histopathology, representing a risk adapted management as already described for the TARGIT A trial [6]. The TARGIT C trial is a phase IV, single arm trial with LEX-IORT during breast conserving surgery for patients above the age of 50 years and again including a risk adapted management (EBRT in addition, if necessary). The TARGIT C trial will start recruitment in 2014. TARGIT BQR is a registry for patients receiving a LEX-IORT boost (standard treatment, no experimental aspect, ClinicalTrials.gov Identifier: NCT01440010). Due to quality assurance of the LEX-IORT boost, this registry is ongoing since 2011 and has recruited 185 patients in 6 centers in Germany until October 2013. While IORT as a boost is accepted as a standard treatment in most countries, e.g. in Germany (S3 guideline [10]), IORT as APBI is not a standard treatment in all countries [11]. Beyond the TARGIT trials with their inclusion and exclusion criteria, different consensus statements exist for patient selection to provide IORT as APBI. Consensus statements for suitable patients are available from the ESTRO (European Society for Radiotherapy and Oncology) [12] and ASTRO (American Society for Radiation Oncology) [13]. The variety of statements and inclusion criteria mark the need for a proper patient selection for IORT especially as APBI. The aim of this analysis was to estimate the eligibility of patients suitable for LEX-IORT as APBI (regarding the ASTRO and ESTRO consensus statements and the inclusion criteria from the TARGIT E and TARGIT C trial) and for LEX-IORT as a boost (regarding the inclusion criteria from the TARGIT BQR registry).

## Methods

**Figure 1:**
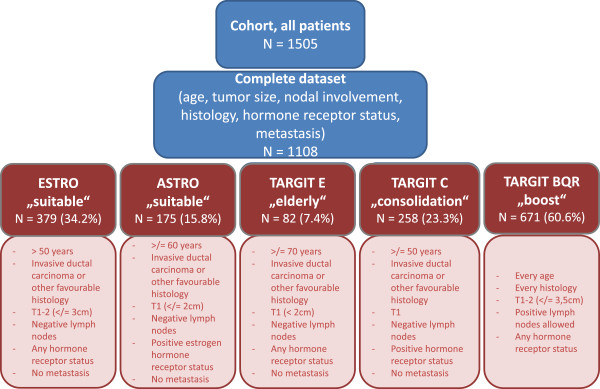
**Eligible patients for IORT: stratification criteria.***Consensus statements:* ESTRO - European Society for Radiotherapy and Oncology and ASTRO - American Society for Radiation Oncology. *Trials:* TARGeted Intraoperative radioTherapy (TARGIT) E – elderly. TARGIT C – consolidation. TARGIT BQR – boost control registry.

Between 01/03 – 12/09, 1505 cases were treated in the breast cancer center of the University Medical Center Mannheim. Primary data of all patients were collected during six years in a database by special staff for tumor documentation. For this analysis available data were screened for complete parameters regarding the ASTRO [13] and ESTRO [12] consensus statements and the inclusion criteria for the TARGIT E (ClinicalTrials.gov Identifier: NCT01299987) [9] and TARGIT C trial and the TARGIT BQR registry (ClinicalTrials.gov Identifier: NCT01440010). Parameters to identify eligible patients are given in detail in Figure 1. Complete data sets for age, tumor size, nodal involvement, final histology type, hormone receptor status and metastases were available in 1108 cases.Percentages were calculated for eligible patients fitting in the predefined groups (ESTRO/ASTRO consensus statements, TARGIT E and TARGIT C trial and TARGIT BQR registry, see Figure 1). Secondly, the percentages of the treated patients with IORT were calculated to estimate the actual rate of treated patients with IORT at a single breast cancer center regarding the ESTRO and ASTRO consensus statements.

The TARGIT E study started recruiting patients after the year 2009 and the TARGIT C study was in preparation at the time point of the present analysis, so no estimates of treated patients within the TARGIT E and C study could be done.

In all patients treated with LEX-IORT, a median of 20 Gy (range, 6 – 20 Gy) was delivered during breast conserving surgery with the INTRABREAM® System (Zeiss Meditec, Oberkochen, Germany) in a single fraction during 20 – 50 minutes, depending on the applicator size. Details of the method were already described elsewhere [14]. LEX-IORT with the INTRABEAM® System has been used since February 2002 at the University Medical Center Mannheim. External beam radiotherapy with 46 – 50.4 Gy was given as a standard treatment with 1.8 – 2.0 Gy per fraction with a dedicated linear accelerator.

## Results

### Eligibility for intraoperative radiotherapy as APBI

**Figure 2:**
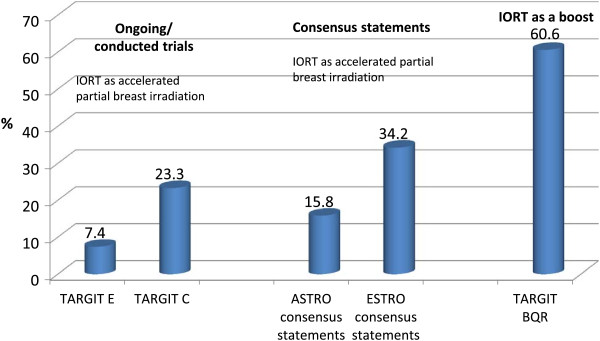
**Eligible patients for IORT as accelerated partial breast irradiation and for IORT as a boost.***Consensus statements:* ESTRO - European Society for Radiotherapy and Oncology and ASTRO - American Society for Radiation Oncology. *Trials:* TARGeted Intraoperative radioTherapy (TARGIT) E – elderly. TARGIT C – consolidation. TARGIT BQR – boost control registry. APBI – accelerated partial breast irradiation.

In general 928 (83.6%) out of 1108 cases were intentionally treated with breast conserving surgery.There were 379 (34.2%) out of 1108 cases eligible for LEX-IORT as APBI regarding the ESTRO consensus statements and 175 (15.8%) cases regarding the ASTRO consensus statements. For the TARGIT E trial, 82 (7.4%) cases and the TARGIT C trial 258 (23.3%) cases were eligible for LEX-IORT as APBI (see Figure 2).

### Eligibility for intraoperative radiotherapy as a boost

As the inclusion criteria are less restrictive for LEX-IORT as a boost, there were 671 (60.6%) cases eligible for an IORT boost (see Figure 2).

### Actually treated patients with intraoperative radiotherapy

According to the consensus statements of ASTRO (79/175; 45.1%) and ESTRO (157/379; 41.4%) about half of the eligible patients were treated with IORT as APBI. From the eligible patients fulfilling the criteria for IORT boost (235/671; 35%) about one third was eventually treated.

## Discussion

Accelerated partial breast irradiation (APBI) for early breast cancer patients is recently used more often as there are several techniques available, showing a good clinical outcome and toxicity rates with usually better cosmetic outcomes [1-8] compared to standard whole breast radiotherapy. The present analysis was conducted to describe eligible patients for IORT with low energy x-rays (=LEX-IORT used as APBI or as a boost) as several consensus statements and different inclusion criteria for studies do co-exist with slightly different criteria. To our knowledge no comparable assessment has been published up to now. Therefore 1108 complete data sets of breast cancer patients (age, tumor size, nodal involvement, final histology type, hormone receptor status and metastases) were first analyzed regarding the eligibility of patients for LEX-IORT as APBI in line with the ESTRO and ASTRO consensus statements and the inclusion criteria for the TARGIT E and C trial. Secondly, eligible patients for LEX-IORT as a boost (TARGIT BQR registry) were identified.

In summary, 18.5% and 34.2% of 1108 cases were eligible for LEX-IORT as APBI in line with the ASTRO and ESTRO consensus statements, respectively. For the TARGIT E trial 7.4% and for the TARGIT C trial 23.3% of all patients were eligible. For LEX-IORT as a boost 60.6% of all patients were eligible.

According to the consensus statements of ASTRO (79/175; 45.1%) and ESTRO (157/379; 41.4%) about half of the eligible patients were actually treated with IORT as APBI. From the eligible patients fulfilling the criteria for IORT boost (235/671; 35%) about one third was eventually treated. Reasons for not receiving IORT were mostly patients with already performed surgery in another department and reasons for planned but not prescribed IORT were already published by Tuschy et al. in 2013. The most reported reasons for omission of a planned IORT were an insufficient skin-tumor distance in 35.1%, an oversized wound cavity in 24.6% and a combination of both in 14% [15].The range of eligible patients for LEX-IORT as APBI ranged from 7.4% (TARGIT E trial) to 34.2% (ESTRO consensus statements). This reflects the variety of different definitions of suitable patients for LEX-IORT as APBI. Having a closer look at the inclusion criteria (Figure 1), age plays a major role in the restriction of eligible patients as a difference of 20 years is between the ESTRO consensus statements (≥50 years) and the inclusion criteria of the TARGIT E trial (≥70 years).

Further 23.3% were eligible for the TARGIT C trial compared to 34.2% eligible patients regarding the ESTRO consensus statements. Indeed, the TARGIT C trial is more restrictive in comparison to the ESTRO consensus statements regarding hormone receptor status and tumor size while both include patients above the age of 50 years. In comparison to the ASTRO consensus statements, the TARGIT C trial differs only as to age: ≥ 50 years (TARGIT C) vs. ≥ 60 years (ASTRO consensus statements) leading to close eligible patient numbers of 23.3% vs. 18.5%, respectively. One may speculate on the role of age per se versus the role of menopausal status which is most likely a biological parameter of higher relevance.

In general, the present analysis shows that patients are highly selected for intraoperative radiotherapy especially used as APBI. Beside patient characteristics, selection criteria for APBI also reflect technical limits of the method itself (e.g. applicator size for IORT with 50 kV/balloon catheter, or number of possible catheters in multi-catheter brachytherapy) or the biological and oncological rationales of tumor treatment (e.g. subclinical tumor invasion around the tumor bed or better prognosis of hormone receptor positive tumors). This trend towards highly selected patients for APBI is in line with certain developments in oncology leading to a more and more personalized treatment of cancer patients. For example, over the last decades mastectomy is no more used for every breast cancer patient but breast conserving surgery with adjuvant radiotherapy is chosen as standard of care, if applicable [11]. Further, the approach of sentinel node biopsy has replaced radical axillary dissection, if applicable [10]. Additionally, personalized systemic treatments including chemotherapy, hormone replacement therapy and targeted therapies with e.g. antibodies are available [10]. In this sense the TARGIT IORT concept with LEX-IORT +/− EBRT is truly a personalized and risk adapted radiotherapy.

The aim is to maximize the patients’ benefits like disease outcome or overall survival and to minimize treatment side effects while not deteriorating quality of life at the same time. To achieve this aim, patient selection is essential. How important such a strict patient selection for IORT is, was shown by Leonardi et al. [16], where the general local recurrence rate of 1822 included patients for IORT with electrons as APBI was 4.1%. In the sub analyses regarding the ESTRO consensus statements (divided into a suitable group, cautionary group and unsuitable group for APBI) the rates for local recurrence were 1.9% in the suitable group, 7.4% in the cautionary group and 7.7% in the unsuitable group (p = 0.001) [16]. The difference was even more significant regarding the ASTRO consensus statements with 1.5% in the suitable group, 4.4% in the cautionary group and 8.8% in the unsuitable group (p = 0.0003) [17]. The rates of local recurrence (1.5 – 1.9%) in the suitable groups after IORT with electrons are in the same range as reported from the TARGIT A trial with 1.2% [6] after LEX-IORT with 50 kV. Within the TARGIT A trial, inclusion criteria were chosen close to the ESTRO consensus statements and secondly a risk adapted management including the addition of EBRT was chosen to face risk factors in final histopathology thus leading to low recurrence rates.

Beyond local control, toxicity and quality of life matters to the patients as mentioned before. After LEX-IORT as APBI and also as a boost, low acute [6,18,19] and long term side effects and especially low chronic skin toxicities were reported [8,20,21]. In general patients with LEX-IORT as APBI were half at risk to develop higher grade late toxicities including fibrosis, breast edema, and edema of the arm, ulceration, hyperpigmentation, telangiectasia, retraction and pain [8]. After LEX-IORT as a boost, fibrosis rates are in range with standard treatments [20,21]. Further quality of life is significantly better in LEX-IORT patients in comparison to patients with whole breast radiotherapy [22,23]. Welzel et al. showed especially less breast and arm symptoms in LEX-IORT patients during an analysis of the TARGIT A patients in a single center [22]. In addition the latest TARGIT A analysis [7] showed a better overall survival in LEX-IORT patients compared to the standard group with whole breast radiotherapy. Vaidya et al. showed also less cardiac events and secondary cancer associated deaths in LEX-IORT patients [7]. These results confirm the low estimated risk for secondary cancers after LEX-IORT shown by Aziz et al. [24]. In context of cost effectiveness, attendance time or room occupation, LEX-IORT seems to be more cost effective and needs less attendance time and room occupation time compared to a standard external beam radiotherapy as shown by Alvarado et al. [25] and Blank et al. [26]. In summary, there are several good reasons from outcome to cost effectiveness to offer intraoperative therapy to eligible patients.

## Conclusions

This analysis shows that patient selection for intraoperative radiotherapy is restrictive, especially for IORT as APBI, and that the TARGIT trials are even more restrictive considering patients for LEX-IORT than the ESTRO and ASTRO consensus statements.

## Competing interests

FW: Radiobiological Research is funded by Zeiss Meditec, Oberkochen, Germany. FW, ES and MS: Travel costs that are linked to the TARGIT trials are covered by Zeiss Meditec, Oberkochen, Germany. All other authors declare that they have no competing interests.

## Authors’ contributions

ES carried out the basic data collection, statistical analysis and drafted the manuscript. DA was mainly involved in data collection and data management. AK, GW, ES, MS, FW were mainly involved in the design of the TARGIT trials and management of patient data. AK, ES, BT, AG, FW and MS were mainly involved in clinical assessment of the patients. All authors, except DA, are involved in the breast cancer center. MS, AG and BT coordinate patients during their hospital stay for intraoperative radiotherapy. All authors read and approved the final manuscript.
